# When context creates uncertainty: experiences of patients who choose rehabilitation as a treatment after an ACL injury

**DOI:** 10.1136/bmjsem-2022-001501

**Published:** 2023-03-22

**Authors:** Ramana Piussi, Rebecca Simonson, Moa Kjellander, Alice Jacobsson, Andreas Ivarsson, Jon Karlsson, Kristian Samuelsson, Eric Hamrin Senorski

**Affiliations:** 1 Health and Rehabilitation, University of Gothenburg Institute of Neuroscience and Physiology, Goteborg, Sweden; 2 Sportrehab Sports Rehabilitation Clinic, Gothenburg, Sweden; 3 Sahlgrenska Sports Medicine Centre, Gothenburg, Sweden; 4 Centre of Research on Welfare, Health and Sport, Halmstad University, Halmstad, Sweden; 5 Swedish Olympic Committee, Stockholm, Sweden; 6 Department of Sport Science and Physical Education, University of Agder, Kristiansand, Norway; 7 Department of Orthopaedics, Sahlgrenska University Hospital, Mölndal, Sweden; 8 Department of Orthopaedics, Institute of Clinical Sciences, Gothenburg, Sweden

**Keywords:** Knee, Rehabilitation, Qualitative

## Abstract

Up to 50% of patients who suffer an anterior cruciate ligament (ACL) injury receive or opt for rehabilitation alone as initial treatment in Scandinavia. Knowledge of whether patients treated with rehabilitation alone after ACL injury are satisfied is lacking. This study aimed to explore the experiences of patients treated with rehabilitation alone after an ACL injury. Fourteen patients (35.9 (19–56) years old) who suffered an ACL injury treated with rehabilitation alone, a mean of 32 months before inclusion, were interviewed. The interview transcripts were analysed using qualitative content analysis with an inductive approach. The experiences of patients treated with rehabilitation after an ACL injury were summarised in one theme: ‘Is the grass greener on the other side? Context characterised by uncertainty’, supported by three main categories and nine subcategories. Uncertainty permeated the context of all levels of knee-related life following ACL injury: (1) in the past, patients felt uncertainty regarding treatment choices, (2) in the present, patients felt uncertainty regarding their physical capacity and knee self-efficacy, and (3) for the future, patients felt uncertainty regarding what might happen. With few exceptions, patients’ experiences after an ACL injury treated with rehabilitation alone are characterised by uncertainty regarding their physical function, psychological impairments and possible future limitation of knee function. Uncertainty is experienced by patients in the past, the present and the future. Patients experience the knee as a symptomatic obstacle and need to adapt the physical activity to the presence of symptoms.

**WHAT IS ALREADY KNOWN ON THIS TOPIC**

- Up to 50% of patients who suffer an anterior cruciate ligament (ACL) injury are treated with rehabilitation alone. There is a knowledge gap regarding the experiences of patients treated with rehabilitation alone after an ACL injury.

**WHAT THIS STUDY ADDS**

- This study reports the experiences of patients treated with rehabilitation alone after an ACL injury. Patients report uncertainty as a major problem in terms of their physical function, psychological impairments, possible future limitation of knee function and persistent knee symptoms.

**HOW THIS STUDY MIGHT AFFECT RESEARCH, PRACTICE OR POLICY**

- The findings of this study are relevant for all clinicians who work with the rehabilitation of patients who suffer an ACL injury in that patient’s uncertainty should likely be addressed in patient–practitioner communication through clear, concise and concrete information about expectations and future outcomes. Further, future studies need to analyse whether uncertainty can be mitigated and how not being uncertain could affect clinical outcomes.

## Introduction

An anterior cruciate ligament (ACL) injury can be treated with rehabilitation alone or with surgical reconstruction and subsequent rehabilitation.^1^ An ACL injury has an incidence of 68.6 injuries per 100 000 individuals yearly in Sweden, of which half are treated with rehabilitation alone.^2^

There is limited research on the outcomes of patients treated with rehabilitation alone.^3^ Recent cohort studies from the Scandinavian registers indicate that patients treated with rehabilitation alone generally report poorer knee function and achieve acceptable knee function to a lesser extent 1, 2, 5 and 10 years after the ACL injury, compared with patients treated with ACL reconstruction and subsequent rehabilitation.^4–6^ Only three randomised controlled trials (RCTs) have analysed the differences in self-reported knee function between the treatment options.^7–9^ The RCTs did not report any clinically relevant differences between the two treatment options concerning self-reported knee function, level of sport or the prevalence of subsequent osteoarthritis (OA) 2–5 years after baseline.^7–10^ However, in these studies,^7–9^ 50%, 38% and 28% of the patients allocated to the rehabilitation alone group opted for subsequent surgery. Therefore, rehabilitation alone as a treatment does not work for all patients who suffer an ACL injury. Historically, patients who suffer an ACL injury and experience knee instability and wish to return to pivoting sports have been offered ACL reconstruction, to stabilise the knee joint and reduce the risk of secondary knee injuries, such as meniscal or chondral injuries.^2^ Consequently, patients treated with rehabilitation alone have been reported to be older and active in less knee-demanding activities compared with patients treated with reconstruction and subsequent rehabilitation.^4–6^ Although the current RCTs do not show clinical differences between treatment options after an ACL injury, there is an apparent selection bias, in which older patients, in everyday practice, are offered rehabilitation alone as a treatment to a greater extent.^6^

According to Sackett^11^ evidence-based medicine involves acting based on three pillars, integrating the clinical experience (1) with the best available research (2) and the patient’s values and preferences (3). Qualitative research provides insight into a patient’s and other stakeholders’ perspectives and plays a major role in integrating patients’ values and preferences into evidence-based medicine. The integration of qualitative research might improve clinical understanding and outcomes^12^ and, allowing patients to share their perception of a certain event, may help researchers and clinicians consider the patients’ experience and perspective.

Collectively, there is an evident knowledge gap concerning how patients treated with rehabilitation alone after an ACL injury perceive treatment outcomes, such as the level of physical activity, the opportunity to participate in physical activity, knee-related quality of life and knee function in activities of daily living. Moreover, knowledge of whether patients treated with rehabilitation alone are satisfied with treatment outcomes is lacking. Therefore, this study aimed to explore the experiences of patients treated with rehabilitation alone after an ACL injury.

## Material and methods

### Study design and participants

Before agreeing to participate in the study, all patients were informed that participation was voluntary, that withdrawal from participation was possible at any time, and that data from the interviews in such cases would be deleted. All the interviews were analysed confidentially. Oral recorded consent was collected at the beginning of each interview.

This study was designed as an interview study where data collected from individual interviews was analysed qualitatively.^13 14^ For transparency, the Consolidated criteria for Reporting Qualitative research (COREQ)^15^ checklist was used to report methodological information.

A convenience sample of patients was recruited through communication with physiotherapists working at sports rehabilitation clinics in Gothenburg, Sweden. Patients who had suffered an ACL injury at least 2 years before the start of this study (1 September 2021) and were treated with rehabilitation alone were eligible for inclusion. Patients of any age or sex were invited. Patients were contacted by telephone, informed about the study and asked whether they were interested in participating. Following a positive response, a meeting was scheduled. To ensure that patients had attempted rehabilitation alone as treatment and were not on the waiting list for ACL reconstruction, a criterion of at least 2 years from ACL injury to the time of the interview was set for inclusion in this study. Patient recruitment continued until no new subcategories emerged from the analysis. As no new data (subcategories) emerged from the analysis, authors deemed to have collected enough data to describe the experience of patients treated with rehabilitation alone after an ACL injury.

Transparency and reflexivity (the process ongoing between the research data and the researchers analysing the data) are important aspects of the qualitative research paradigm. Therefore, we provide information about the research team’s background and reflect on how our experiences might influence our interpretation of the collected data. The first author, a male (RP), and the second author, a female (RS), are both experienced sports physiotherapists. The third (MK) and the fourth (AJ) authors are two female physiotherapists with a special interest in sports injury rehabilitation. In terms of the other authors, one (KS) is an orthopaedic surgeon (professor), and one (AI) is a researcher and sports psychology consultant (professor), both with extensive experience in the research field. The sixth author (JK) is a male senior orthopaedic surgeon (professor) who largely contributed to the development of science in knee traumatology. The senior author (EHS) is a male senior physiotherapist (associate professor), active within the clinical and the research field. The first and senior authors were responsible for the study’s conception and execution. Three authors (RS, MK and KS) have suffered ACL injuries treated with reconstruction.

The authors’ presumptions were developed based on clinical experience working with patients treated with rehabilitation alone after an ACL injury. Presumptions include the general hypothesis that patients who choose rehabilitation alone as treatment typically display faster recovery in early rehabilitation but long-term physical symptoms (ie, pain) and psychological (ie, fear of movement) impairments, compared with their counterparts treated with early ACL reconstruction. Presumptions of psychological aspects were that patients who choose rehabilitation alone as a treatment would not perceive rehabilitation as long but would experience more negative emotions at the time of interviews. Moreover, the authors’ presumptions included the notion that ACL reconstruction is a better choice for patients active in sports who display knee instability after an ACL injury. These presumptions were discussed extensively by the authors throughout the analysis process. There was no personal relationship between any of the patients who participated in the study and the study’s authors.

### Patients and public involvement statement

Three authors (RS, MK and KS) have had an ACL injury treated with ACL reconstruction and provided clinical and patient perspectives in the conception of the study and the development of the interview guide. Neither patients nor the public were involved in recruitment to or the conduct of the study or were asked to assess the burden of participating.

### Data collection

An interview guide was created through discussion between authors. Examples of open-ended questions included ‘Seen from a knee-related perspective, how would you say your life is today?’; or ‘How has the injury and rehabilitation process changed your life?’. The semistructured interview guide can be found in online appendix A.

10.1136/bmjsem-2022-001501.supp1 Supplementary data

Data were collected by the first (RP) and second authors (RS) between October and November 2021 in Gothenburg (Sweden). Due to the COVID-19 pandemic, all interviews were performed digitally (via ZOOM web-based application). No other person besides the interviewer and the informants was present at the interview. During the interviews, no field notes were taken, and the interviewers provided none of their assumptions in order not to bias the participants. Interviews were recorded via the ZOOM recording function. The mean length of the interviews was 21 (range 10–35) min, and records were transcribed verbatim. Transcripts were not sent to the participants for corrections or comments to minimise the patient burden, and the validity of this method has been questioned.^16^ After 14 interviews, no other new aspects emerged and patient inclusion ended.

### Data analysis

We used an interpretive/constructivist epistemological approach to capture the multiple realities, descriptions, and experiences of ACL injured populations, and acknowledges that the researcher also plays a role in the construction of the data, that is, there is no separation between the researcher and the data. Since going through such a complicated process (ie, injury and rehabilitation) is a highly individual experience, we believe the choice of individual interviews to be justified.

An inductive approach using qualitative content analysis based on the framework developed by Graneheim and Lundman^13 14^ was used to analyse the data. The first author was primarily responsible for the data analysis process but continuously triangulated data among the other authors who controlled the coding and categorisation process and where presumptions were continuously discussed. The analysis was performed by RP, RS, MK, AJ, AI and EHS, and extensive discussions took place to increase the trustworthiness in relation to the results. Any disagreement was resolved in consensus after discussing with the senior author. Transcripts were first read thoroughly to obtain a general understanding of the data. Second, meaningful units were extracted and shortened into condensed meaningful units. The condensed meaningful units were then abstracted and coded. Codes addressing similar categories were grouped into subcategories, and subcategories were then grouped into main categories. The manifest content in the data was presented as main categories, while the latent content was summarised into a theme. After grouping categories into subcategories, the transcripts were read again and subcategories were validated against the transcripts to ensure that data were not missed or erroneously included. The analysis process is iterative and was performed back and forth through discussions between the authors involved in the analysis.

**Figure 1:**
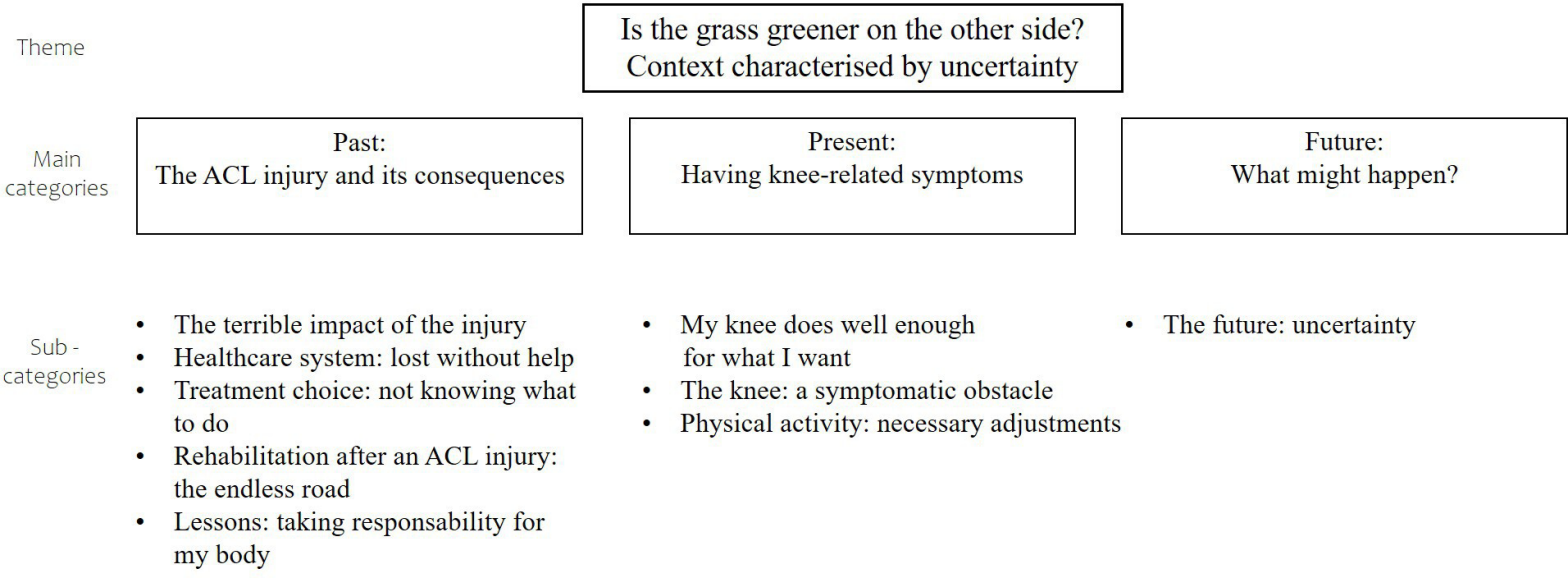
Overview of the theme, main categories and subcategories generated from data analysis. ACL, anterior cruciate ligament.

Qualitative content analysis was first developed as a branch of quantitative analysis, with the aim of counting how often certain words appeared in a text.^17^ After analysis, to show the preponderance of codes found, based on the historical roots of the method,^17 18^ we chose to add a quantitative representation of codes comprised within each subcategory. Accordingly, in figure 1, the total number of codes in every subcategory is presented in a spider diagram.

To increase the transparency of the qualitative research, table 2 presents an example of codes and the grouping in subcategories and main categories starting from patients’ quotes.

## Results

Fourteen interviews with 14 patients were conducted. Table 1 presents the patients’ demographics.

**Table 1:** Demographics for the 14 included participants

Sex	
Male (%)	6 (43)
Age, years	
Mean	35.9
Median; range	37; 19–56
Time from ACL injury and interview, months	
Mean	32
Median; range	32; 24–44

ACL, anterior cruciate ligament.

One theme, supported by three main categories and nine subcategories, was derived from the collected data. Figure 1 presents an overview of the theme, main categories and subcategories.

The experiences of patients treated with rehabilitation alone after an ACL injury were summarised in one theme: ‘Is the grass greener on the other side? Context characterised by uncertainty’. Uncertainty permeated every level of knee-related life following an ACL injury (1) in the past, patients felt uncertainty about treatment choices and possible outcomes, (2) in the present, patients felt uncertainty about their physical capacity and knee self-efficacy and (3) for the future, patients felt uncertainty about what might happen (subsequent injury, level of future physical activity) and if it might have been better to choose ACL reconstruction.

Patients experienced uncertainty in the period following the ACL injury due to the inability of healthcare providers to address all the questions the patients had. Patients felt that healthcare providers were prone to suggest a ‘wait and see’ approach, which was perceived as insecurity and an inability to make a proper treatment decision. When patients attempted to confront healthcare providers to obtain further answers, no clear information was received, and uncertainty resulting from the ACL injury and the subsequent period persisted for patients into the present. When offered the opportunity for delayed ACL reconstruction, patients did not perceive this option as the best possible treatment but rather as a way for healthcare providers to let time go by and hope for spontaneous symptom resolution.

**Figure 2:**
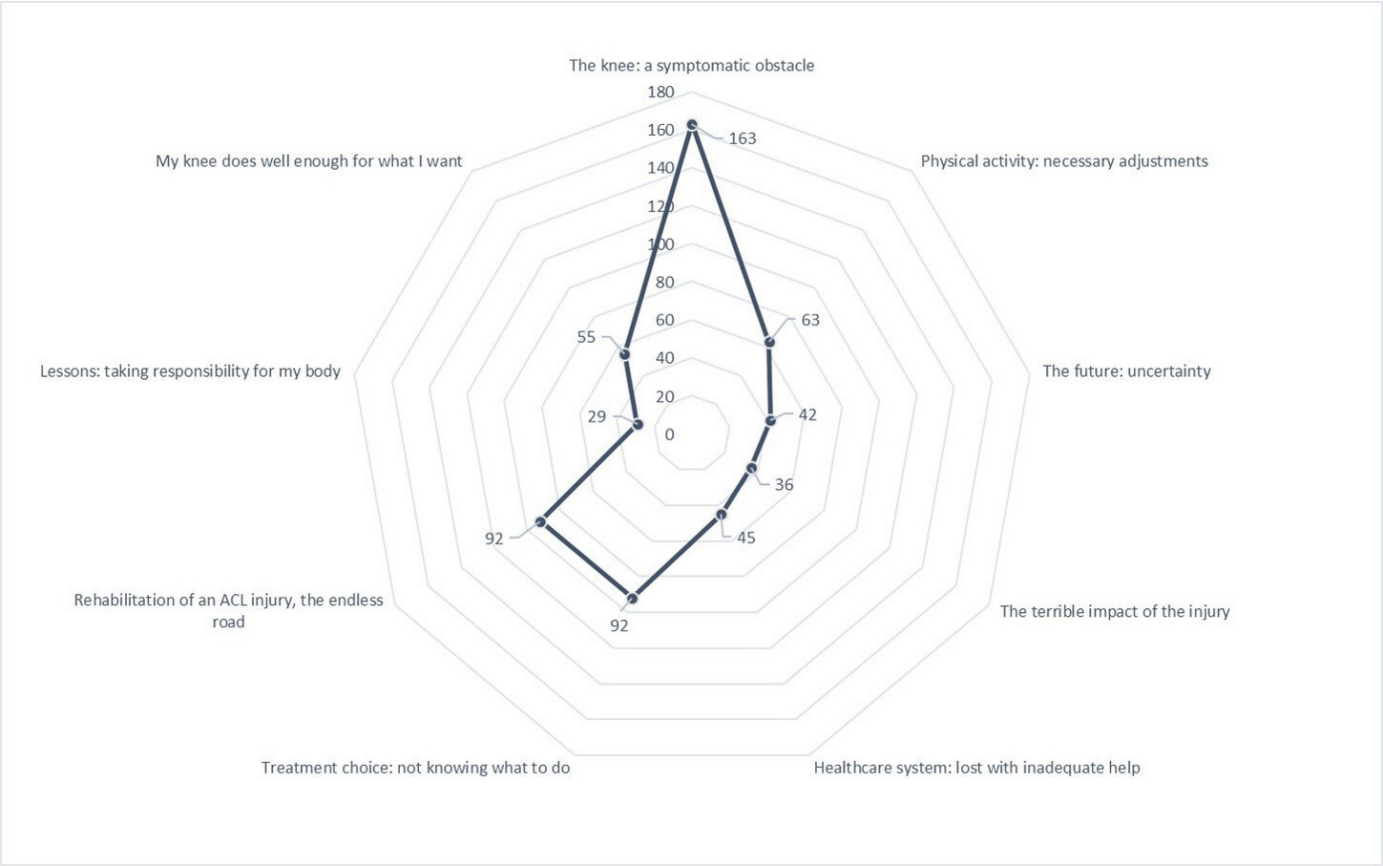
Visual representation of the number of codes in each subcategory. ACL, anterior cruciate ligament.

The analysis resulted in nine subcategories, where codes were unevenly distributed among the main categories. Figure 2 illustrates the quantitative preponderance of codes in the nine subcategories.

### Main category: past: the ACL injury and its consequences

Patients described their ACL injury as a life-changing event that entailed major changes, such as changes in their primary physical activity and perceiving their bodies as fragile. Some patients reported suffering from setbacks such as knee pain or an inability to trust their knee, which still tormented the patients from the ACL injury and rehabilitation process. On the other hand, other patients reported that they did not suffer from any consequences of the ACL injury; therefore, they stated that the ACL injury and rehabilitation process resolved without major adaptations in life.

#### The terrible impact of the injury (36 codes)

After suffering the ACL injury, some patients stated that they did not know what the ACL injury meant. In contrast, other patients described feeling shocked and almost ‘depressed’ when they realised what the ACL injury meant in terms of limitations and severity of symptoms. Table 2, Q1.

**Table 2:** Quotes from interviews for each subcategory

Main category	Subcategory	Code	Quote
Past: the ACL injury and its consequences	The terrible impact of the injury	Impaired body Terrible Difficult to understand	Q1: I love to use my body. I am not an elite athlete, but… I love to move(…), and this (feeling the body as impaired) is perhaps the worst thing. That… feeling my body is impaired. It was terrible. It has been enormously difficult to understand.
	Healthcare system: lost without help	Nobody told me I did not understand Did not get information I Had to book myself Not understand my injury Very tough programme produced symptoms	Q2: I did not get any information about… this is something that I first understood long after the injury. But I should not have walked on my injured leg. But nobody told me. I did not understand how badly my leg was injured.(…)so we went in (to the hospital) to get some crutches. And I still did not receive any information. I had to book an appointment with a physiotherapist by myself. Then I came there and the physiotherapist was not able to determine what type of injury I had.(…)so, I got a very tough rehabilitation programme, which gave me a lot of knee symptoms.
	Treatment choice: not knowing what to do	The surgeon decided I had no choice Reading literature Consulted with a physiotherapist Surgery would not be better	Q3(A): But he (the surgeon) said: “This is not a case for surgery”. There was no discussion, I had no choice. Q3 (B): I had been reading some of the literature on the subject and then consulted my physiotherapist… and… as I understood, there was no belief that surgery would result in a superior outcome.
	Rehabilitation of an ACL injury: the endless road	It was a shock Could not contact muscles I could not move my knee Nice to come back to the gym Going to the gym gave motivation Quick differences From injured to strong Got excited	Q4 (A): And then I remember… I remember it was like a shock. I was unable to use my muscles.(…)it was a simple task: extend the knee. I remember I was staring at my knee, but it just wouldn’t extend. Q4 (B): It was so nice to come back (to the gym)! And at the same time… it (coming back to the gym) gave me more motivation… To do the old exercises… I noticed the difference very quickly… From injured to strong. I got excited!
	Lessons: taking responsibility for my body	Turning point when I listened to my body Did not trust healthcare providers I looked at my body from a different perspective I had to take responsibility for my treatment	Q5: The turning point in the rehabilitation was when I started listening to my body. I did not blindly trust doctors or physiotherapists. I took a step back and looked at my body and soul… What do I need? I had to learn to take responsibility for my treatment.
Present: having knee-related symptoms	My knee does well enough for what I want	Fully active Play my sport Play without problems	Q6: I am fully active. I play my sport in division one without any problem.
	The knee: a symptomatic obstacle	Knee is not as before I do not accept it Sense of weakness in the leg Cannot trust the leg Not trusting the leg limits me	Q7: The leg… it is not as it was before. And I do not want to accept it any more. The leg stays together. I know inside my head my leg can support my torso… But… I have a sense of weakness inside there. I cannot trust my leg as I did before. I feel this limits me from doing what I love to do.
	Physical activity: necessary adjustments	I do not perform as before Run slower Take it easy because of the knee	Q8: … on the other hand, when you think about jogging, I am not where I was before (the injury). So, when I am out running now, we are talking about five, six kilometres, at a totally different pace compared with before the injury… But… it is still jogging, if we can call it that…but I consciously take it easy in order not to strain my knee too much.
Future: what might happen?	The future: uncertainty	Keep training the rest of my life Knee needs training Do not know what might happen if I do not train	Q9: Yes, I think I will have to keep on training for the rest of my life… My knee needs training. But, in the future, when I am old… if I feel I am not able to train… I don’t know what will happen then. We shall see.

ACL, anterior cruciate ligament.

#### Healthcare system: lost with inadequate help (45 codes)

The encounter with the healthcare system was experienced as complicated and troublesome. Some patients said they were very dissatisfied with the level of competence of the healthcare providers and were frustrated that they had to wait months for their diagnosis. The patients’ experiences of the inability and incapability of the healthcare system to take care of their injury, as well as shortcomings in the information provided by the healthcare system, helped to create uncertainty about the patients’ knees and outcomes. Table 2, Q2.

#### Treatment choice: not knowing what to do (92 codes)

Some patients stated that they were not given the opportunity to choose treatment for their ACL injury but felt compelled by the physician to undertake rehabilitation alone as a treatment. Some patients actively decided to give treatment with rehabilitation alone after ACL injury a chance. Regardless of the reason behind the treatment choice, patients searched for information on the internet, asked friends or relatives, consulted with responsible physiotherapists and read scientific publications on the subject to obtain more information about their injury and treatment. Table 2, Q3A+B.

#### Rehabilitation of an ACL injury: the endless road (92 codes)

Although patients felt that rehabilitation was important to promote benefit and regain control of their bodies, rehabilitation was perceived as time-consuming and demanding physically and psychologically. Patients experienced difficulty realising that their bodies did not function as they had before the ACL injury. For patients who completed rehabilitation, evaluation, sympathetic physiotherapists dedicated to the patient, loving and supportive significant others and self-discipline were described as factors contributing to success. However, not all patients said they had the mental strength to maintain motivation and complete rehabilitation to regain acceptable knee status. Table 2, Q4A+B.

#### Lessons: taking responsibility for my body (29 codes)

For some patients, learning to ‘listen to my body’ was described as the turning point in rehabilitation, when the symptoms started to resolve. Patients described ‘listening to my body’ as granting the body some rest when training generated symptoms. An additional way to ‘listen to my body’ was to plan social activities based on what the knee could withstand and not on what patients wished to do. Some patients said that the ACL injury made them humble and forced them to take responsibility for their bodies. Learning to accept the new situation was described as a great challenge and, importantly, as a major lesson. Table 2, Q5.

### Main category: present: having knee-related symptoms

In some exceptional cases, the ACL-injured knee did not feel injured at all, and life continued as before the ACL injury. However, for most patients, the knee was seen as an impediment, a constant reminder of the ACL injury, more or less symptomatic every day. Patients experienced uncertainty about the capability of their knee to perform physical tasks such as running, jumping or hiking. Patients reported not being able to trust their knee. The inability to feel confidence in the ability of their knee was believed to have a negative impact on knee-related self-efficacy.

#### My knee does well enough for what I want (55 codes)

Few patients said they were grateful for the recovery of their knee and were satisfied with the treatment outcome with rehabilitation alone after an ACL injury. One patient returned to their preinjury level of sport (competitive ice hockey) without symptoms. It was common for the patients who were satisfied with the outcome to say that their knee did well enough to match the demands they placed on their knee. Table 2, Q6.

#### The knee: a symptomatic obstacle (163 codes)

For most of the patients interviewed, their ACL-injured knee was seen as an obstacle in everyday life, which contributed to a feeling of being limited and having a low belief in what the knee was able to withstand. The perception of the knee as an obstacle was due to symptoms such as pain, swelling and excessive tiredness or stiffness. Physical symptoms were said to entail psychological concerns, such as feelings of uncertainty, fear, reduced knee-related self-efficacy and horrible thoughts about the knee, ultimately limiting the patients. Limitations generated by knee-related symptoms affected the patients, expressed socially as the inability to participate in social activities to the same extent as they had before the ACL injury. Moreover, some patients experienced unhappiness, dissatisfaction and felt their body was disabled, and they wished that the ACL injury had never happened. Patients thought about their knee daily and could not trust the knee’s ability to perform physical tasks. Consequently, some patients reported not having the courage to work out or even use their knee due to fear of a second knee injury to either knee. Table 2, Q7.

#### Physical activity: necessary adjustments (63 codes)

Due to the knee-related symptoms, patients were forced to adapt their everyday life and physical activities to less knee-demanding physical activities. Adaptations in everyday life included feeling forced to use lifts instead of stairs or avoiding exposure to perceived injury risks such as running for the bus. Patients did not report adapting physical activity with the help of healthcare professionals but rather arriving at the adaptation through their own ‘trial and error’. Examples of adaptations to physical activities included changed running style, reduced running pace, frequency and distance. Table 2, Q8.

### Main category: the future and what might happen

For some patients, uncertainty about the future included not knowing whether their perceived knee symptoms would ever disappear. Uncertainty was perceived when patients thought about what would happen when they were no longer able to train their knee. Some patients felt that they were caught in a loop of constant training necessary to minimise knee symptoms.

#### The future: uncertainty (42 codes)

Concerns described by patients as permeating the past and present experiences were also reported in relation to the future. Patients experienced uncertainty about the status of their knee in the future and whether it might have been different if they had undergone ACL reconstruction. Patients reported fear of the potential onset of knee OA. Table 2, Q9.

## Discussion

The main findings in this qualitative study comprised one theme, reflecting three main categories. Overall, the time after an ACL injury treated with rehabilitation alone was characterised by uncertainty. Patients did not receive clear answers to questions and felt unable to trust their knee and did not know what the future might hold. Most patients experienced persistent knee symptoms, whereas a few patients could return to a normal life without knee impairment.

### Theme: is the grass greener on the other side? context characterised by uncertainty

The theme of this study, ‘Is the grass greener on the other side? Context characterised by uncertainty’, interprets the state of mind of the interviewed patients when they reflected on the past, present and future. Several patients were worried about their knee function after injury and what consequences the ACL injury could have in the long term. Early reconstruction after ACL injury entails superior patient-reported outcome compared with both rehabilitations alone and later ACL reconstruction.^5 6^ These findings are inconclusive, since there are studies reporting no differences between patients treated with rehabilitation alone or reconstruction in patient-reported outcomes in the long term (10–20 years) after ACL injury.^19 20^ On the other hand, patients treated with ACL reconstruction can report persistent symptoms and dissatisfaction with treatment.^21^ A lack of information from the healthcare system with regard to treatment options resulted in the absence of proper guidance and patients, therefore, experienced being confused, frustrated and uncertain. Patients in this study reported wondering whether or not a decision about late ACL reconstruction could improve their knee function and reduce limitations. Clinically, patients might interpret one of the two treatment options, rehabilitation alone or ACL reconstruction, or the lack of clear information, as one treatment leading to a complete resolution of symptoms or impairments. Clinicians should stay up to date with the literature be aware that there is no superior option at this stage,^22^ and treatment decisions should be made with patient preferences and needs in mind.

Uncertainty was a central aspect of patients’ experiences of rehabilitation alone as a treatment after an ACL injury. Uncertainty refers to a psychological state of ignorance, but, rather than mere ignorance, to the ‘conscious awareness or experience of ignoring something’.^23^ To be aware of ignoring something, that is, being uncertain, has the potential to influence the emotions and thoughts a patient might have in relation to a certain event in which the ignorance is taking place. There can be different sources of uncertainty, but two important sources related to the findings in this study are (1) probability, which arises from the unpredictability or indeterminacy of the future, and (2) ambiguity, which arises from limitations in the reliability, credibility or adequacy of the information.^24^ Uncertainty is mostly regarded as being negatively charged.^23^ In response to uncertainty, patients can experience negatively charged affective feelings, such as anger, anxiety or sadness, and uncertainty during an emotional event has been reported to make unpleasant events more unpleasant.^25^ From a healthcare perspective, negatively charged affective feelings can influence treatment outcomes and patient satisfaction.^26^ One specific outcome that can be affected is the patient’s confidence in their ability to perform a physical task (self-efficacy).^27^ Experiencing uncertainty might lead to patients having lower levels of knee-related self-efficacy, where lower levels of self-efficacy have been linked to a lower level of performed physical activity.^28^ Not being confident in one’s self-efficacy might lead to patients being afraid of the moment and possibly developing kinesiophobia, which can negatively affect rehabilitation after ACL injury.^29^ Taken together, patients’ uncertainty can lead to a cascade of negative affects, and the healthcare system’s inability to provide patients with adequate and credible information can negatively influence treatment outcomes. Consequently, the healthcare system needs to improve the amount, quality and credibility of information with regard to treatment options and outcomes given to patients who suffer an ACL injury.

In Sweden, where this study was performed, patients who suffer an ACL injury are commonly assessed by a physiotherapist and eventually referred to an orthopaedic surgeon. Physiotherapists treating patients after ACL injury might feel uncertain since evidence on treatment after an ACL injury is under constant development and far from certain.^30^ As research advances, it is up to researchers and healthcare professionals (eg, treating physiotherapists, surgeons) to create and update clinical practice guidelines to provide patients with clarity about their treatment options.

### Main category: past: the ACL injury and its consequences

Patients described their first encounter with healthcare providers as unconvincing. The overall experience was a lack of professional assertiveness, and patients were instead met with vagueness regarding the outcomes of the different treatment choices. Several patients experienced not being a part of the treatment decision-making process. In contrast, some patients reported reading scientific studies and consulting with friends to make a proper decision. For some patients, the rehabilitation was tough and mentally challenging. Social support, intended as a forum to discuss and share experiences and resources of informational (education) or emotional type, has been previously stated to play an important role for patients to take part in successful rehabilitation.^30 31^ In line with this statement, patients in this study explained that the support and presence of physiotherapists were important to maintain rehabilitation when motivation was low. It is, therefore, important for physiotherapists who work with patients who suffer an ACL injury to provide proper support during rehabilitation.

### Main category: present: having knee-related symptoms

Previous RCTs^7 8^ have determined that there is no significant difference in terms of self-reported knee function, level of physical activity or the prevalence of subsequent OA 2–5 years after an ACL injury between patients treated with rehabilitation alone and patients treated with ACL reconstruction and subsequent rehabilitation. In this study, there were occasional exceptions, where patients reported that they did not suffer from knee symptoms and that their knee function was good enough for what was needed. On the other hand, many patients said that their knee was a constant reminder of their injury and that symptoms were a daily struggle. In line with previous research, patients treated with rehabilitation alone experienced poorer self-reported knee function.^4^ Physical and psychological stress can affect the training and rehabilitation of the knee.^32^ Uncertainty and undetailed guidance reflect hesitation and doubt on the part of healthcare providers and might increase patients’ psychological stress. It is plausible that this uncertainty will remain with the patient throughout the treatment process and that the healthcare professional’s hesitant behaviour fuels the psychological impairments. It is important that the healthcare professional has a great knowledge of rehabilitation alone as a treatment after an ACL injury, has confidence in the treatment provided to the patient and is clear when providing information and expectations relating to injury and treatment. Knowing how to take care of patients’ psychological state of mind after an ACL injury is also important. The great preponderance of codes in the subcategory ‘The knee, a symptomatic obstacle’ highlights that many patients do not have a satisfactory present knee function. Therefore, future studies to understand whether clearer information changes patients’ uncertainty and which subgroup of patients who suffer an ACL injury might benefit from rehabilitation alone as a treatment are needed.

### Main category: the future and what might happen

The uncertainty experienced by patients in the past and the present was perceived in relation to the future. Patients experienced not knowing what could happen in the future, whether they would ever regain proper knee function and whether they would be able to participate in physical activities without limitations imposed by their knee. Further uncertainty was experienced about the future’s unpredictability, specifically in the possible development of knee OA. Whether OA would occur cannot be predicted by healthcare providers, and, as a result, uncertainty about unpredictable outcomes can probably not be answered. However, a recent umbrella meta-analysis showed that an ACL injury increases the risk of developing knee OA.^33^ Moreover, surgical treatment does not appear to reduce knee OA prevalence compared with rehabilitation alone as treatment.^33^ With regard to the presence of uncertainty for future knee OA, the implementation of a virtual knee-health programme aimed to minimise impact of knee OA for people at risk of post-traumatic OA after a sport-related knee injury, has been reported to satisfy an unmet need.^34^ Accordingly, the uncertainty for future knee OA has been highlighted in other geographical setting than those included in this study. Taken together, patients end up feeling the same way as they initially experienced treatment from the healthcare system, ‘wait and see’; hoping for symptom resolution but experiencing uncertainty.

### Methodological discussion/limitations

Qualitative research plays an important role in bridging the gap between research and practice, as patients’ voices are given a chance to be included to create new understanding. When including the patient as the main stakeholder, quantitative and qualitative research is needed to explore various aspects of health-related issues.^12^

Qualitative content analysis can provide access to each participant’s subjective construction of a certain event, and was deemed suitable to realise our aim and individual interviews were, therefore, chosen as a data collection method. The description according to Graneheim and Lundman^13 14^ implies that data are derived via an interaction between the researcher, the participants and the analysed text. Researchers work, therefore, through own bias and preconceptions during the analysis. In order to account for the interaction between researchers and data, own bias and preconceptions were discussed in the method section, and the six authors participated in the analysis via regular meetings, where findings were continuously discussed. According to the description formulated by Graneheim and Lundman^13^ trustworthiness can be further divided into credibility, dependability and transferability. To establish credibility, researchers need to accurately describe the research participants. Accordingly, patient demographics were reported in table 2, and each involved research background and demographics were briefly discussed in the method, according to COREQs. To provide credibility in the analytical process, examples of the analytical process are provided in table 1, ranging from codes to main categories. Dependability refers to the certainty of how the analytical process has been carried out and the stability of data over time. To ensure dependability, the interview guide was worked on before the study started and not changed afterwards. The goal with this project was not to achieve transferability but rather to capture experiences of individuals who rarely have the opportunity to share their experience, since outcomes after rehabilitation alone as treatment after ACL injury has not been as thoroughly studied as outcomes after ACL reconstruction. Consequently, the transferability of the results should be interpreted with caution.

Another possible limitation could be the brevity of some interviews. Despite time not merely reflecting the richness of the interview, the length of the interview can influence the amount of information collected. In our sample, two interviews lasted 10 min, one lasted 14, and all the other interviews lasted well above 20 min. Common for the short interviews was that informants were rather satisfied with their knee function in relation to the patient’s current knee demands.

Lastly, we used a convenience sample, which might not entirely reflect the variety of experiences of patients treated with rehabilitation alone after ACL reconstruction. Future studies could apply more selective inclusion criteria to produce specific results for certain subgroups of patients who suffer an ACL injury and are treated with rehabilitation alone.

### Summary

With few exceptions, patients’ experiences after an ACL injury treated with rehabilitation alone are characterised by uncertainty regarding their physical function, psychological impairments and possible future limitation of knee function. Uncertainty is experienced by patients in the past, the present and the future. Patients experience the knee as a symptomatic obstacle and need to adapt their physical activity to the presence of knee symptoms.

## Data Availability

Data are available on reasonable request.
