# Supplementary material for: When context creates uncertainty: experiences of patients who choose rehabilitation as a treatment after an ACL injury
Source: BMJ Open Sport Exerc Med. 2023 Mar 22;9(1):e001501. doi: 10.1136/bmjsem-2022-001501 (PMC10040041; doi:10.1136/bmjsem-2022-001501)
Supplement: Supplementary data [file bmjsem-2022-001501supp001.pdf]

Interview guide for study When context creates uncertainty: experiences of patients who choose rehabilitation as a treatment after an ACL injury

- Can you please tell me how you injured your ACL?
- How physically active are you today?
- (in case of playing sports): what does your sport mean to you?
- Seen from a knee related perspective, how is your life today?
- What do you feel about your knee?
- Why did you choose not to treat you ACL injury surgically?
- How did you make that choice?
- What did you base your decision on?
- How has your ACL and rehabilitation process affected your life?
- Could you tell me about your experiences of rehabilitation?
- Which meaningful happenings have occurred during your rehabilitation?
- How do you feel about having an ACL injured knee today?
- Which consequences from the injury do you experience in your daily life?
- Could you tell me about your knee function today?
- What do you feel about the treatment you have had (rehabilitation)?
- Whan would you have changed if you could go back in time?
- Do you have any proposition for improvement?
- How do you think about your knee and the future?
- How would you summarise it is to live with an ACL injured knee?
- Is there anything you would like to add?
